# Evolutionary algorithm search for connectivity patterns conducive to bursting in respiratory neural networks

**DOI:** 10.1186/1471-2202-15-S1-P25

**Published:** 2014-07-21

**Authors:** Daniel T Robb, Maya Shende, Peter Griffin, Natalia Toporikova

**Affiliations:** 1 Department of Mathematics, Computer Science and Physics, Roanoke College, Salem, VA 24153, USA; 2 Department of Biology, Washington and Lee University, Lexington, VA 24450, USA

## 

The respiratory neural network in the pre-Botzinger complex of the ventrolateral medulla controls and flexibly maintains the breathing rhythm, coordinating network-wide bursting to signal the inspiratory phase of the breath. Interestingly, however, the connectivity of the network by which it achieves this coordination is still a subject of active research. We search for connectivity patterns which yield high performance in a measure of network bursting, using an evolutionary algorithm (EA)-based approach, in networks of leaky integrate-and-fire (LIF) and conductance-based model (CBM) neurons. In LIF networks, the connectivity patterns of bursting networks are characterized by regularities in several network statistics: closeness centrality(CC), betweenness centrality (BC) and out-degree (OD) distributions. We find additionally that intentionally selecting for these statistical regularities in CC, BC and OD leads to steady improvement in the network bursting measure. We examine the extent to which these trends in network statistics are present in well-adapted networks of CBM neurons without intrinsically bursting neurons, finding that the regularities in BC and OD are also visible. We also present initial results on the statistical regularities in networks of CBM neurons with intrinsic bursters.

**Figure 1:**
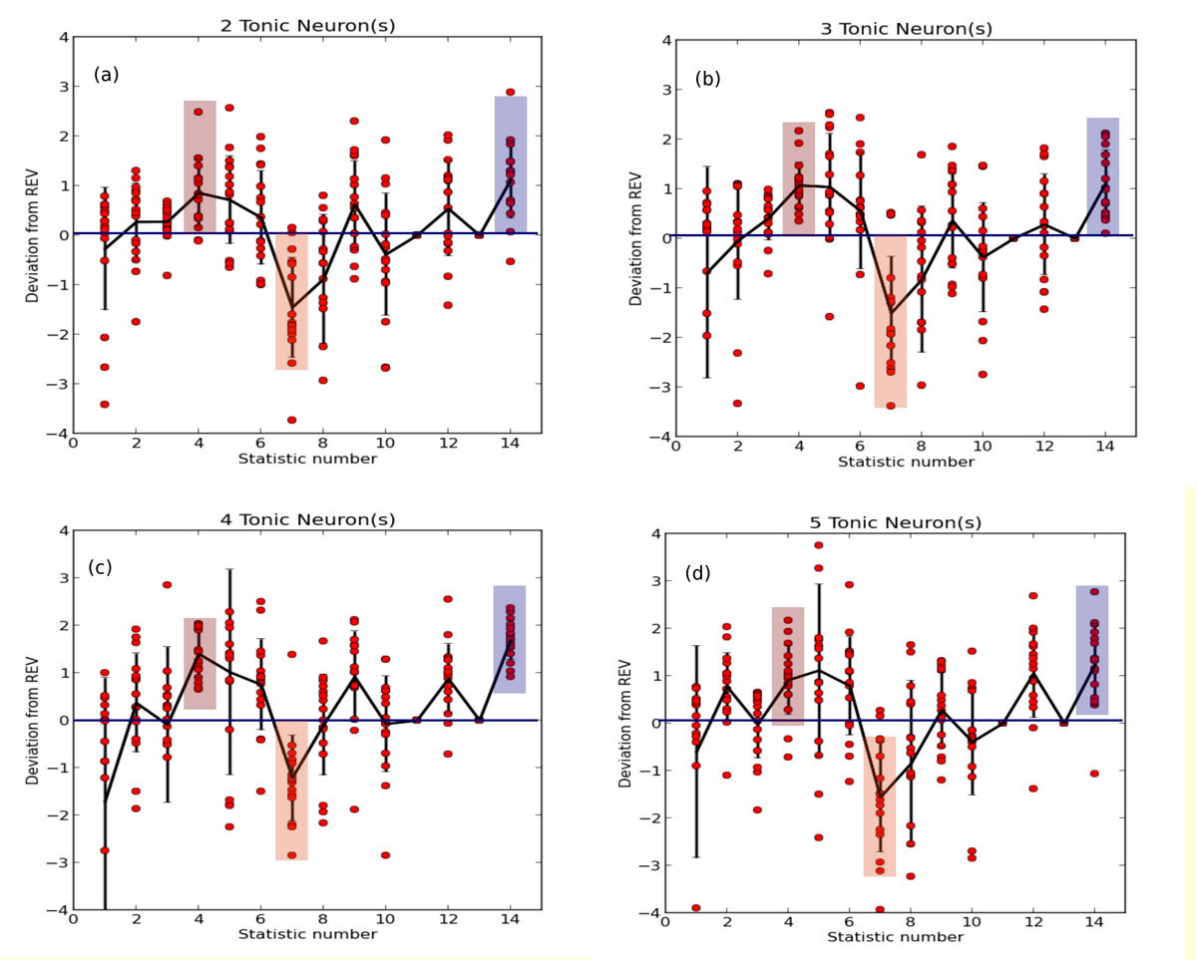
illustrates the regularities in closeness centrality (CC), betweenness centality (BC) and out-degree (OD) distributions in well-adapted networks of LIF neurons. In well-adapted networks with (a) 2, (b) 3, (c) 4 and (d) 5 tonically spiking neurons present (of 20 total neurons), these three network statistics show statistically significant differences from random networks with the same average connectivity. Note that in the figure, CC is statistic number 4, BC is statistic number 7, and OD is statistic number 14.

